# A methodological approach to age estimation of the intra‐puparial period of the forensically relevant blow fly *Calliphora vicina* via Fourier transform infrared spectroscopy

**DOI:** 10.1111/mve.12748

**Published:** 2024-08-02

**Authors:** Luise Thümmel, Johannes Tintner‐Olifiers, Jens Amendt

**Affiliations:** ^1^ Goethe‐University Frankfurt, University Hospital, Institute of Legal Medicine Frankfurt am Main Germany; ^2^ Department of Aquatic Ecotoxicology, Faculty of Biological Sciences Goethe University Frankfurt am Main Germany; ^3^ Academy of Fine Arts, Institute of Natural Sciences and Technology in the Art Vienna Austria

**Keywords:** ATR‐FTIR, *Calliphora vicina*, forensic entomology, pupal age estimation

## Abstract

Estimating the age of immature blow flies is of great importance for forensic entomology. However, no gold‐standard technique for an accurate determination of the intra‐puparial age has yet been established. Fourier transform infrared (FTIR) spectroscopy is a method to (bio‐)chemically characterise material based on the absorbance of electromagnetic energy by functional groups of molecules. In recent years, it also has become a powerful tool in forensic and life sciences, as it is a fast and cost‐effective way to characterise all kinds of material and biological traces. This study is the first to collect developmental reference data on the changes in absorption spectra during the intra‐puparial period of the forensically important blow fly *Calliphora vicina* Robineau‐Desvoidy (Diptera: Calliphoridae). *Calliphora vicina* was reared at constant 20°C and 25°C and specimens were killed every other day throughout their intra‐puparial development. In order to investigate which part yields the highest detectable differences in absorption spectra throughout the intra‐puparial development, each specimen was divided into two different subsamples: the pupal body and the former cuticle of the third instar, that is, the puparium. Absorption spectra were collected with a FTIR spectrometer coupled to an attenuated total reflection (ATR) unit. Classification accuracies of different wavenumber regions with two machine learning models, i.e., random forests (RF) and support vector machines (SVMs), were tested. The best age predictions for both temperature settings and machine learning models were obtained by using the full spectral range from 3700 to 600 cm^−1^. While SVMs resulted in better accuracies for *C. vicina* reared at 20°C, RFs performed almost as good as SVMs for data obtained from 25°C. In terms of sample type, the pupal body gave smoother spectra and usually better classification accuracies than the puparia. This study shows that FTIR spectroscopy is a promising technique in forensic entomology to support the estimation of the minimum post‐mortem interval (PMI_min_), by estimating the age of a given insect specimen.

## Graphical abstract

- Prediction of the intra‐puparial age of *C. vicina* was possible based on ATR‐FTIR‐absorption spectra.
- For age estimation, the full spectral range from 3700 to 600 cm^−1^ and the use of support vector machines resulted in the most accurate predictions.
- Both sample types tested, the freeze‐dried and homogenised pupal body and the oven‐dried puparium, resulted in accuracies of more than 85%.

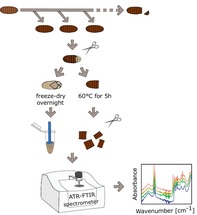

## INTRODUCTION

Blow flies are often used for an estimation of the minimal post‐mortem interval (PMI_min_), as they are usually the first insects to colonise a body after death (Amendt et al., 2011; Catts & Goff, 1992). Using immature specimens from the corpse or its surroundings, forensic entomologists can estimate the PMI_min_ within the first days to weeks after death by determining the age of the oldest specimen (Amendt et al., 2004, 2007, 2011; Buchan & Anderson, 2001). While the age of larvae can be determined by measuring their length or calculating the time taken to reach certain stages in their development (e.g., post‐feeding or pupariation), the age estimation of the intra‐puparial form is not that easy, because all morphological changes are not visible from the outside of the puparium. As blow flies spend up to 50% or more of the total developmental time in this stage (Marchenko, 2001), an accurate estimation of the intra‐puparial age is of great importance for forensic entomologists. Until now, estimating the intra‐puparial age still relies on the further breeding of sampled specimens at a known constant temperature (Amendt et al., 2007, 2011) and thus requires up to several days until a statement of the age can be made. Additionally, it relies on living specimens, which are not always available due to, for example, being killed by the crime scene technicians. Assessing the histological and morphological changes (Davies & Harvey, 2013; Wang et al., 2018; Zajac & Amendt, 2012) can be subjected to observer bias and difficult to quantify if not combined with more complex and cost intensive methods like micro‐computed tomography (CT) (Martín‐Vega et al., 2017; Nur Aliah et al., 2019; Richards et al., 2012) or optical coherence tomography (Brown & Harvey, 2014). Furthermore, gene expression analysis (Ames et al., 2006; Boehme et al., 2013, 2014; Hartmann et al., 2021; Shang, Yang, et al., 2023; Tarone & Foran, 2011; Wang et al., 2018; Zajac et al., 2015; Zehner et al., 2009), protein expression analysis (Fu et al., 2023) and the analysis of cuticular hydrocarbon (CHC) profiles (Bosorang et al., 2017; Shang, Feng, et al., 2023; Shang, Yang, et al., 2023) allow for quantification as well, but they can be expensive and are not yet sufficiently analysed with regard to their variation. For this reason, there is a need for a rapid and cost‐effective technique which can be used either as a complement to the results of the above mentioned methods or as a stand‐alone technique.

Fourier transform infrared (FTIR) spectroscopy is based on the ability of molecules to absorb electromagnetic radiation with their functional groups and turn it into specific molecular vibrations (Alkhuder, 2022; Berthomieu & Hienerwadel, 2009). The resulting infrared bands can then be used to characterise the biochemical structure of the tested material. Coupled to an attenuated total reflection (ATR) unit, FTIR spectroscopy can also be used to examine samples directly in the solid state, which means it can be a nearly non‐destructive method and reduce the amount of sample preparation. Moreover, the generation of infrared spectra is a very rapid method, and as FTIR spectroscopy does not require any consumables or chemicals, it is also very cheap. Due to all of these advantages, FTIR spectroscopy has become a powerful tool in forensic and life sciences. It has been used, for example, to characterise different cell types and monitor carcinogenesis (Baker et al., 2014; Movasaghi et al., 2008), to screen for diseases like severe acute respiratory syndrome coronavirus 2 (SARS‐CoV‐2) (Barauna et al., 2021) and to detect bacteria in blood samples (De Sousa Marques et al., 2014; Maquelin et al., 2003). Additionally, it is commonly used for the identification of paint and synthetic fibres (Stuart, 2021) and to analyse and age‐grade biological traces like hair or body fluids (Alkhuder, 2022; Lin et al., 2017).

By monitoring biochemical changes in the body during the decomposition process, FTIR spectroscopy has been used to distinguish between causes of death (Wang, Tuo, et al., 2017; Zhang et al., 2023) or to estimate the PMI (Huang et al., 2008; Ke et al., 2012; Wang, He, et al., 2017; Wang, Zhang, et al., 2017; Zhang et al., 2017, 2020). It has also already been used to identify or discriminate insects (Barbosa et al., 2018; González Jiménez et al., 2019; Pickering et al., 2015). Moreover, this method is becoming increasingly popular in other fields, such as archaeometry, as it can be used to date various materials, such as wood or paper (Tintner, 2021). Therefore, it is a promising technique in forensic entomology research to support the estimation of the PMI_min_ by age grading insect specimens found at a crime scene (Jales et al., 2021). FTIR spectroscopy creates high‐dimensional data that require machine learning tools to be analysed. These algorithms can handle large datasets and have become a standard tool in biological research and medicine in the past years (Ching et al., 2018; Greener et al., 2022). One can generally distinguish between two variations of machine learning, supervised and unsupervised methods. Whereas supervised methods require the data to be labelled in order to train a model that can be used to predict the label of an unseen data point, unsupervised methods, such as clustering, group data points based on their similarities (Ching et al., 2018; Greener et al., 2022).

*Calliphora vicina* Robineau‐Desvoidy is a synanthropic blow fly distributed throughout the Holarctic region (Rognes, 1991). It was shown to be active all year round in Germany (Fremdt & Amendt, 2014; Lutz et al., 2019) and is the second most abundant blow fly species colonising cadavers (Lutz et al., 2021). This regular association with human cadavers makes the species very important in forensic entomology.

The aim of this study was to obtain first‐time developmental reference data for the changes in the absorption spectra throughout the intra‐puparial period of *C. vicina* via FTIR spectroscopy. For this, *C. vicina* was bred at two constant temperatures, and individuals were killed every second day from the onset of pupariation until adult emergence. As, to our knowledge, no other study has compared which part of the specimens, that is the pupal body itself or the former cuticle of the third larval instar, is best suited for predicting the age of the specimens, both sample types were compared in this study. Furthermore, different wavenumber regions of the absorption spectrum were used to train two different machine learning models to classify the intra‐puparial age of *C. vicina*. This study serves as a proof‐of‐concept that there are sufficient measurable changes in FTIR‐absorption spectra to assist forensic entomologists in estimating the PMI_min_ and will suggest methodological approaches for future studies.

## MATERIALS AND METHODS

### Fly breeding

Adult flies of a pre‐existing laboratory colony that was established with *C. vicina* collected in the area of Frankfurt/Main, Germany were kept at room temperature (approximately 22°C) and 12:12 L:D in cages measuring 35 × 26 × 26 cm and were provided with sugar and water *ad libitum*. Once a week, flies were provided with beef liver as a protein source and oviposition substrate.

### Developmental study

For oviposition, beef liver was placed in the fly cages for 3 h, and the resulting eggs were transferred into climate chambers (Binder KB 53, E3.1) without illumination at 20°C and 25°C, respectively. After hatching, larvae were placed in three groups of approximately 150 larvae (per temperature) on 100 g of minced meat (half beef/half pork) in a plastic cup (i.e., 3 × 150 larvae at 20°C and 3 × 150 larvae at 25°C). Each cup was placed in a container measuring 12 × 12 × 8 cm. Containers were checked every 24 h and as soon as the larvae reached their post‐feeding state, migrating larvae were separated into smaller groups of 50 and placed in separate containers with small animal litter as a pupation medium. This further separation was necessary because it was observed that overcrowding in the containers delays the onset of pupariation (Whiting, 1914). As soon as white pre‐pupae formed, they were separated and the containers monitored at shorter time intervals. When more than 50 individuals pupariated within 8 h, they were separated from the rest and the day of pupariation was marked as day 0.

Four specimens were randomly selected every other day (starting from day 1 after pupariation) until the first emergence of adult flies and killed by freezing at −20°C without any prior manipulation. This resulted in six time points (day 1, 3, 5, 7, 9 and 11) for *C. vicina* reared at 20°C and four time points (day 1, 3, 5, 7) for flies reared at 25°C. This procedure was replicated five times, with one oviposition event per week, resulting in 20 specimens per age/sampling day and temperature regime. As the adult flies of one of the replicates (at 20°C) already started to emerge during the sampling on day 11, only a total of 16 specimens were collected for this day.

### Sample preparation

To investigate which part of the specimens is more suitable for age classification using FTIR‐absorption spectra during the intra‐puparial period, each specimen was divided into two subsamples: the pupal body, hereafter referred to as ‘pupa’ and the former cuticle of the third larval instar, called ‘puparium’.

Frozen specimens were rinsed with distilled water to remove any traces of small animal litter and dried on filter paper. The anterior part of the puparium was then opened with tweezers, and the pupa was carefully removed from the puparium without rupturing the specimen. Empty puparia were dried at 60°C for 5 h and cut into smaller fragments to enable an adequate placement with the ATR unit on top of the crystal (see below). Drying before measurement was necessary to avoid distortion of the infrared spectra due to absorption peaks caused by water. This resulted in more uniform spectra across all samples. Pupae were placed on a sterile petri dish and freeze‐dried overnight. Dried pupae were subsequently transferred into 1.5 mL micro‐centrifuge tubes and ground with a pestle. All prepared samples were stored at room temperature and measured within two days after preparation.

### Collection of infrared spectra

Absorption spectra were recorded on a JASCO FT/IR‐4200 spectrometer equipped with a Ge/KBr beam splitter and a DLaTGS (deuterated L‐alanine doped triglycine sulphate) detector, coupled to a PIKE Technologies GladiATR accessory. Measurements were performed with a resolution of 4 cm^−1^ and 32 scans in the range of 4000–400 cm^−1^ per sample (Durak et al., 2022; Tintner et al., 2020). Five spectra were recorded from each ground pupa, while the former abdominal segments 1–6 of the puparium of each specimen were measured four times with the outside facing the crystal. Background spectra were recorded before the placement of each specimen and automatically subtracted from the spectra.

### Data analysis

Spectral data were analysed in R (version 4.3.1) with RStudio (version 1.4.1103) (R Core Team, 2020). As the 4000–3700 and 600–400 cm^−1^ regions showed a lot of background noise, these wavenumber regions were removed in the first step of pre‐processing. To normalise the data and to reduce the effects of light scattering and variations in sample thickness, all spectra were pre‐processed using the standard normal variate (SNV) (Barnes et al., 1989). The four (puparia) or five (pupae) spectra from each sample were first visually checked for outliers, then averaged to reduce the noise and eventually considered as a single spectrum, each. All analyses were performed separately for the spectral data of the puparia and pupae. Each dataset was randomly split into a training and a test set (75% / 25%) with each age equally represented in both sets.

To distinguish the wavenumber regions that give the best classification results, the spectra were subsequently divided into four groups: full spectrum (3700–600 cm^−1^), the biological fingerprint region (1800–900 cm^−1^), the methyl/methylene region (3000–2800 cm^−1^) and a combination of both aforementioned regions (3000–2800 and 1800–900 cm^−1^). Two supervised machine learning models were trained and tested to classify the age of *C. vicina* pupae: Random forests (RF) and support vector machines (SVM). In addition, multi‐dimensional scaling (MDS), based on the proximities calculated by the RF models (using squared distances in a Euclidean space), was used as an unsupervised model to reduce the dimensionality of the dataset and to visualise the distances between samples.

#### Random forests

To construct the trees, the package randomForest (version 4.7–1.1) was used (Liaw & Wiener, 2002). The algorithm splits the training dataset into several new ones of equal size by randomly selecting subsamples of the original dataset. As this approach results in data that are not included in the training of the algorithm (building the tree), the ‘unknown’ data are called ‘out of bag’ (OOB) data. These OOB data are then presented to the trees to estimate the accuracy of the model (OOB estimate). Accuracy is defined as the number of all correctly classified cases out of the total number of cases. For each RF, the best number of trees was chosen by visualising the error rates, and the optimal amount of variables that were tried at each split was tested using the tuneRF option of this.

#### Support vector machines

To build models based on SVMs, the R package e1701 (version 1.7.14) was used (Meyer et al., 2023). Models were built using a linear kernel and the regularisation parameter (C) was adjusted for each model. To determine the accuracy of the model, a 10‐fold cross‐validation was performed.

Additionally, the accuracy of each model was calculated based on correctly predicted cases as a percentage of all classifications considering the test set data. To visualise the comparison between the two sample types and the different models for the respective wavenumbers tested, confusion matrices were created based on the predicted and actual results of the test set data by using the caret package (version 6.0.94) (Kuhn, 2008). In the matrix, each cell value represents the number of samples that were classified accordingly. While the columns represent the actual results, the rows show the predicted results.

## RESULTS

**FIGURE 1:**
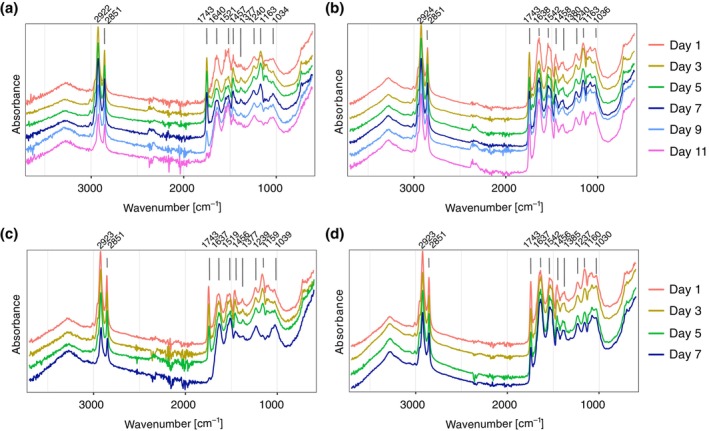
Average Fourier transform infrared (FTIR) absorption spectra of the pupae and puparia of *Calliphora vicina* of all collected spectra per age and temperature in the range of 3700–600 cm^−1^. Absorption maxima are labelled and, to allow better comparison of spectra from specimens of different ages, shifted along the *y*‐axis. (a) Puparia, 20°C; (b) pupae, 20°C; (c) puparia, 25°C; (d) pupae, 25°C.

At a constant temperature of 20°C, the first adult *C. vicina* emerged 12 days after pupariation, while at a constant temperature of 25°C, the first flies emerged after 8 days. A comparison of the FTIR‐absorption spectra of averaged spectra of *C. vicina* reared at constant 20°C and 25°C showed that all samples, irrespective of their age, rearing temperature and preparation method showed two distinct maxima in the methyl/methylene band region at ~2923 and 2851 cm^−1^ (Figure 1, Table 2). The second wavenumber region which showed several absorption maxima and could therefore be of interest for the prediction of the time since pupariation (= the age of the pupa/puparia), is the biological fingerprint region at 1800–900 cm^−1^. Eight absorption maxima were identified in this region, independent of age, sample type and rearing temperature (for identification of the maxima see Table 1). While specimens reared at 20°C showed a relatively high infrared absorbance intensity at 1743 cm^−1^ from day 1 to day 7, which can be associated with a symmetrical C=O stretch linked to lipids, this intensity decreased in 9‐day‐old specimens and was almost absent in puparia of 11‐day‐old specimens (Figure 1a). A similar pattern was observed for *C. vicina* reared at constant 25°C. Here, the oldest specimens (7‐days‐old) also showed almost no absorbance maximum at the corresponding wavelength for the sample type ‘puparium’ (Figure 1c), while the absorption maximum is relatively higher for pupae of the same age (Figure 1d). Pupae of every age also show a relatively high absorbance in the Amide I region (1640–1637 cm^−1^), which is associated with proteins, whereas this absorbance maximum appeared to be lower for puparia (Figure 1; Table 2), irrespective of the rearing temperature.

**TABLE 1:** Identification of absorption maxima of the obtained Fourier transform infrared (FTIR) spectra (Durak et al., 2022; González Jiménez et al., 2019; Nopp‐Mayr et al., 2020; Schwanninger et al., 2004; Shang, Feng, et al., 2023).

Wavenumber [cm^−1^]	Assignment
3292–3269	NH‐stretch^a^
2924‐2922	C—H stretch (asymmetric)
2851	C—H stretch (symmetric)
1743	C=O stretch (symmetric)
1640–1637	C=O stretch, Amide I
1542–1519	C=N stretch and N—H bend, Amide II
1458–1456	C—H bend
1385–1377	C=O stretch
1240–1239	O—H and C—H bend
1163–1159	C—O stretch
1039–1030	C—O stretch

^a^ The NH‐stretch coincides with a stretch of bound OH‐groups in the area of ~3600–3100 cm^−1^, which are therefore not listed separately here.

**TABLE 2:** Accuracy of RF and SVM classification models based on internal validation (OOB estimation or 10‐fold cross‐validation [cv]) and test set data for all wavenumber regions tested, sample types and both rearing temperature settings.

	Wavenumbers [cm^−1^]	Sample type	Accuracy [%]			
			RF		SVM	
			OOB estimate	Test set	10‐fold cv	Test set
20°C	3700–600	Puparia	73	17.2	85.9	96.5
		Pupae	88.4	44.8	87.2	86.2
	1800–900	Puparia	58.8	17.2	54.1	55.2
		Pupae	77.9	31	80.2	62.1
	3000–2800	Puparia	50.6	24.1	35.3	51.7
		Pupae	57	31	45.3	41.4
	3000–2800 and 1800–900	Puparia	57.7	20.7	54.1	65.5
		Pupae	81.4	27.6	77.9	65.5
25°C	3700–600	Puparia	86.4	75	84.7	90
		Pupae	85	95	93.3	95
	1800–900	Puparia	71.2	50	76.3	65
		Pupae	81.7	85	81.7	75
	3000–2800	Puparia	69.5	60	67.8	50
		Pupae	61.7	70	50	60
	3000–2800 and 1800–900	Puparia	72.9	40	71.2	65
		Pupae	86.7	85	80	80

Abbreviations: CV, cross‐validation; OOB, out of bag; RF, random forests; SVM, support vector machine.

**FIGURE 2:**
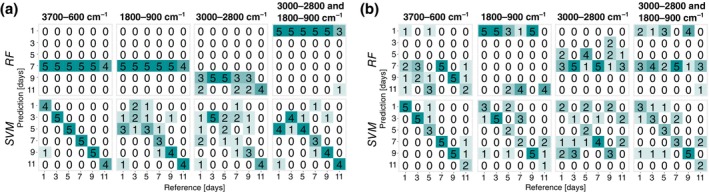
Confusion matrices of the performance of the two machine learning age classification models (random forests [RF] and support vector machine [SVM]) on the test set data for four different wavenumber regions. Classification was based on spectral data collected from puparia (a) or pupae (b) of *Calliphora vicina* reared at constant 20°C.

**FIGURE 3:**
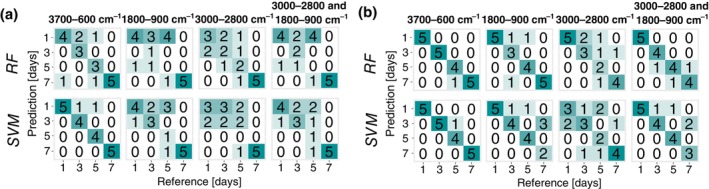
Confusion matrices of the performance of the two machine learning age classification models (random forests [RF] and support vector machine [SVM]) on the test set data, for four different wavenumber regions. Classification was based on spectral data collected from puparia (a) or pupae (b) of *Calliphora vicina* reared at constant 25°C.

The prediction of the age of specimens showed a high variability in the classification accuracy depending on the range of the spectral wavenumber regions and the sample type. At 20°C, the RF models created using training spectra from pupae were consistently better than those built from training spectra obtained from the puparium. The best RF model based on the full spectral range of 3700–600 cm^−1^ of measurements of the pupae yielded an accuracy of 88.4% when considering the OOB estimate. However, RF models performed poorly, with 44.8% being the highest accuracy when subjected to completely new spectral data from the test set (Table 2; Figure 2). SVMs showed also the highest classification accuracy based on cross‐validation of the training set of the full spectral range of 3700–600 cm^−1^ of measurements of the pupae. When the model was presented with completely new data (test set), the accuracies even exceeded the cross‐validation results (Table 2). Here, the highest accuracy based on the test set data was 96.5%, and the full spectral range was obtained for spectra obtained from the puparia. SVMs were better at predicting the age of *C. vicina* pupae reared at 20°C than RF models, irrespective of the wavenumber region, when presented with test data (Figure 2). For 25°C, the RF models performed better than at 20°C, with smaller differences between the accuracies based on the OOB estimate and the test set data (Table 2; Figure 3). Similar to results obtained from specimens reared at 20°C, the full spectral range from 3700–600 cm^−1^ resulted in the best classification accuracies, with a strong preference for the ‘pupae’ sample type (95% accuracy) for both, RF and SVM, when presented with test set data (Table 2; Figure 3).

**FIGURE 4:**
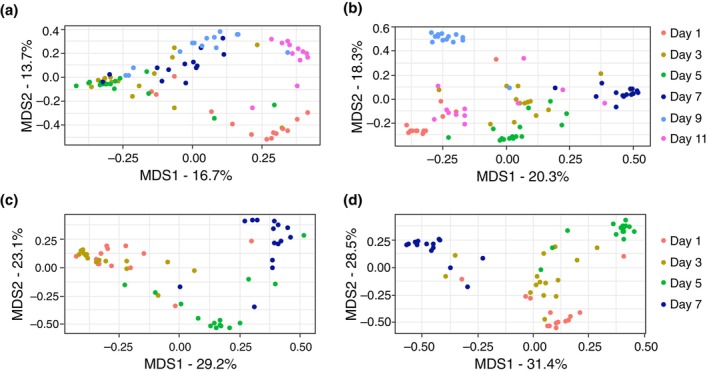
Multi‐dimensional scaling (MDS) ordination of Fourier transform infrared (FTIR) absorption spectra from 3700 to 600 cm^−1^ of the age (in days) of *Calliphora vicina*'s pupae and puparia based on squared distances in an Euclidean space. (a) Puparia, 20°C; (b) pupae, 20°C; (c) puparia, 25°C; (d) pupae, 25°C.

Based on these results, MDS was performed on the favourable wavenumber region (full spectral range of 3700–600 cm^−1^). While a clustering of samples of the same age, especially day 1 and day 11, was already visible based on data from the puparia (Figure 4a), the spectral data obtained from measurements of the pupae showed an even better separation of samples of different ages (Figure 4b). The first MDS axis covered 20.3% of the variation and showed good separation of days 1, 5 and 7, while day 9 was clearly separated by the second MDS axis, which accounted for 18.3% of the variation in the training dataset. For *C. vicina* reared at 25°C, the reduction of dimensionality of the training dataset also resulted in a better separation of samples when considering the pupae rather than the puparia (Figure 4c,d), with the first MDS axis covering 31.4% of the variation and the second axis covering 28.5%.

## DISCUSSION

The development time from pupariation to adult emergence took approximately 11–12 days at 20°C and 8 days at 25°C, which is within the range of previous studies (Marchenko, 2001; Zajac et al., 2018).

In addition to confirming development times, it is much more important that the current study accurately estimated not only for the first time the intra‐puparial age of *C. vicina* using FTIR‐absorption spectra but also showed promising results for correct age classification in combination with machine learning algorithms. This means an additional method of age determination in forensic entomology, which is not having a high risk of intra‐observer bias while not being expensive and laborious.

Chitin and protein, the two main components of the insect cuticle (Fraenkel & Rudall, 1940, 1947), and the lipid layer covering it, including CHCs (Blomquist et al., 1987; Lockey, 1988), are reflected in the FTIR‐absorption spectra of the puparia. While the absorption maxima in the carbohydrate 1240–1030 cm^−1^ wavenumber region may represent the chitin (González Jiménez et al., 2019), the maxima associated with lipids may be related to the CHCs present on the puparia, where they are thought to prevent desiccation of the insect or infestation by bacteria or fungi (Blomquist et al., 1987; Wang et al., 2022; Yang et al., 2022).

Gibbs and Crowe focused exclusively on the methyl−/methylene region (3000–2800 cm^−1^) to visualise biophysical changes in insect CHCs using FTIRs (Gibbs & Crowe, 1991). It has previously been confirmed that the CHC composition changes during larval and intra‐puparial development in blow flies and flesh flies (Alotaibi et al., 2021; Bosorang et al., 2017; Moore et al., 2013; Shang, Feng, et al., 2023; Shang, Yang, et al., 2023; Sharma et al., 2021; Xu et al., 2014); this was the wavenumber region that resulted in the worst predictions. These changes therefore do not appear to be striking or regular enough in IR‐absorption spectra.

As proteins, lipids and carbohydrates are also the main biomolecules in cells, it is not surprising that the absorption spectra of the homogenised pupal body show an almost similar FTIR‐absorption pattern. Although previous studies have described the selection of the fingerprint region (1800–900 cm^−1^) for predicting the age of homogenised flesh fly pupae (Shang, Feng, et al., 2023; Shang, Yang, et al., 2023) or for estimating the PMI via biochemical changes inside the cadaver (Wang, He, et al., 2017; Wang, Tuo, et al., 2017; Wang, Zhang, et al., 2017; Zhang et al., 2017, 2020, 2023), this region, which provides information on changes in carbohydrates (e.g., chitin) and proteins, did not perform as well as the full spectral range in the current study. The most accurate results were obtained by including data from the full spectral range from 3700 to 600 cm^−1^, which was also shown previously (Zhang et al., 2024). Furthermore, when considering the most important wavenumbers that were used to build the trees in the RF models, these wavenumbers were distributed throughout the full spectral range (data not shown). Similarly, important information for species and age classification of mosquitos was also found throughout the full FTIR‐spectral range (González Jiménez et al., 2019). Due to the large differences between the accuracy of the OOB estimates and the prediction of the RF models when presented with new data (the test set), it appears that RF models tend to overfit the spectral data more than the SVMs. Therefore, SVM seem to be the better choice in this case.

Both sample types, puparia and pupae, resulted in high classification accuracies. However, while most of the correct classifications of the test set data were obtained using the full spectral range and SVM based on spectra obtained from the puparia at 20°C, the pupae at 25°C resulted in higher accuracies. In addition, the MDS ordination of both sample types resulted in slightly better clustering of different age categories based on measurements of the pupae.

Overall, the handling of the homogenised pupae at the spectrometer was easy and resulted in smooth spectra of similar intensities with little background noise. In contrast, although the preparation of the puparia is slightly easier and needs less drying time, the placement of the puparia on the crystal and the application of pressure with the ATR unit required more experience as puparia may break due to their curvature. This can also result in loss of the sample once measured and reduces the number of repeate measurements that can be made on each sample. Furthermore, the furrowed surface of the puparia does not allow for a total exclusion of air between the sample and the crystal, which is possibly one of the reasons why the obtained spectra are not as smooth as for the powder of the homogenised pupal tissue. Therefore, we suggest using homogenised pupal tissue for future age estimation studies based on FTIR spectroscopy. However, if a freeze dryer is not available, puparia can be used as the spectra obtained are suitable too.

The differences in the performance of the machine learning models with respect to the two rearing temperatures are very noticeable, as the age estimation of *C. vicina* pupae reared at 25°C was more accurate than at 20°C. As we had the same time intervals of two days for the measurements at both temperatures, one reason for this different performance may be that more changes took place within 48 h at the higher temperature of 25°C (e.g., between day 3 and 5) than at 20°C, resulting in more easily distinguishable spectra. However, by including accumulated degree hours (ADH) in the next developmental study and killing the pupae reared at different temperatures at defined ADH, this hypothesis could be tested. More classification overlap was also shown for mosquito ageing when comparing models built with data collected on consecutive days compared with data from every two to three days (González Jiménez et al., 2019). The authors also hypothesised that this change in model performance is due to an overlap in the features used to build the models when measurements are taken on consecutive days (González Jiménez et al., 2019).

Spectroscopic methods such as FTIR spectroscopy, near‐infrared spectroscopy and hyperspectral imaging are powerful tools in (forensic) entomology (Jales et al., 2021; Johnson & Naiker, 2020; Wang et al., 2016). They are not only sufficient for species and age identification of pests (Dowell et al., 1999; Durak et al., 2022; González Jiménez et al., 2019; Omucheni et al., 2023; Perez‐Mendoza et al., 2004) but also for the detection of parasitized eggs (Nansen et al., 2013). However, for FTIR spectroscopy to find its way into the courtroom, further research is needed.

As the police and forensic pathologists are often not trained in the proper sampling and storage of insect evidence, the models need to be tested to see if storage liquids, such as ethanol or formalin, and extended storage periods in these liquids alter the FTIR‐absorption spectra and thus interfere with the age estimation of samples.

We showed that age classification is possible, but regression models for an accurate age estimation of the intra‐puparial period of blow flies should be tested in future approaches combined with larger sample sizes and shorter sampling intervals. Moreover, future studies should not only focus on more temperature regimes but also include fluctuating temperatures, which were already shown to result in slightly different spectral patterns (Shang, Yang, et al., 2023). As it was previously shown that CHC profiles differ between populations of *C. vicina* in Europe (Moore et al., 2022), this might also be the case for FTIR spectra. Therefore, it needs to be verified whether regionally different populations of blow flies result in slightly different absorption spectra, which may result in worse age predictions. However, machine learning models trained with near‐infrared spectra obtained from lab reared mosquitos were still able to sufficiently predict the age of mosquitos in wild populations (Milali et al., 2018). To create more stable models, the sample size needs to be increased, thereby allowing the usage of additional machine learning algorithms such as neural networks (LeCun et al., 2015). By an increased sample size, intra‐age differences like sex specific differences will also be decreasing, as those differences might have affected the classification accuracy (Dowell et al., 2005; Trabalon et al., 1992).

## CONCLUSION

FTIR spectroscopy can be successfully used in combination with machine learning algorithms to estimate the age of *C. vicina* intra‐puparial forms. However, with the current approach, it is not possible to estimate the intra‐puparial age to the day. Thus, a combination of different methods, such as micro‐CT analysis of the same specimen, followed by the dissection of the specimen and a description of morphological landmarks before performing a FTIR spectroscopy analysis, would allow the age to be determined as accurately as possible, while minimising observer bias. Therefore, the application of such multi‐method studies should be the focus of future studies.

## AUTHOR CONTRIBUTIONS

**Luise Thümmel:** Conceptualization; formal analysis; investigation; methodology; visualization; writing – original draft. **Johannes Tintner‐Olifiers:** Conceptualization; methodology; writing – review and editing. **Jens Amendt:** Conceptualization; project administration; writing – review and editing.

## CONFLICT OF INTEREST STATEMENT

The authors declare no conflicts of interest.

## Data Availability

The data that support the findings of this study are available from the corresponding author upon reasonable request.
